# Exploring Perceptions and Needs of Mobile Health Interventions for Nutrition, Anemia, and Preeclampsia among Pregnant Women in Underprivileged Indian Communities: A Cross-Sectional Survey

**DOI:** 10.3390/nu15173699

**Published:** 2023-08-24

**Authors:** Avishek Choudhury, Yeganeh Shahsavar, Krishnendu Sarkar, Murari Mohan Choudhury, Ashish D. Nimbarte

**Affiliations:** 1 Industrial and Management Systems Engineering, Benjamin M. Statler College of Engineering and Mineral Resources, West Virginia University, Morgantown, WV 26506, USA; ys00022@mix.wvu.edu (Y.S.);; 2 NSHM Knowledge Campus, Kolkata 700053, India; 3 Network for Enterprise Enhancement and Development Support (NEEDS), Deoghar 814143, India

**Keywords:** maternal health, digital intervention, ehealth, human factors, sustainable development goals, technology acceptance model, health belief model

## Abstract

According to the National Family Health Survey of 2021, about 57% of women aged 15–49 in India currently suffer from anemia, marking a significant increase from the 53% recorded in 2016. Similarly, a study conducted in southern India reported a 32.60% prevalence of preeclampsia. Several community-based initiatives have been launched in India to address these public health challenges. However, these interventions have yet to achieve the desired results. Could the challenges faced by traditional healthcare interventions be overcome through a technological leap? This study assesses pregnant mothers’ perceptions regarding mobile health interventions for managing anemia and preeclampsia. Additionally, the study captures their health awareness and knowledge. We conducted a survey with 131 pregnant mothers in three underserved villages in Jharkhand, India. Statistical analysis was conducted using the SEMinR package in R (Version 2023.06.0), utilizing the non-parametric partial least squares-structural equation modeling. We found that every household had at least one smartphone, with the respondents being the primary users. The main uses of smartphones were for calling, messaging, and social media. A total of 61% of respondents showed interest in a nutrition and pregnancy app, while 23.66% were uncertain. Regarding nutritional knowledge during pregnancy, 68.7% reported having some knowledge, but only 11.45% claimed comprehensive knowledge. There was a considerable knowledge gap regarding the critical nutrients needed during pregnancy and the foods recommended for a healthy pregnancy diet. Awareness of pregnancy-related conditions such as anemia and preeclampsia was low, with most respondents unsure of these conditions’ primary causes, impacts, and symptoms. This study serves as a critical step towards leveraging technology to enhance public health outcomes in low-resource settings. With the accessibility of mobile devices and an apparent willingness to utilize mHealth apps, compounded by the pressing need for improved maternal health, the impetus for action is indisputable. It is incumbent upon us to seize this opportunity, ensuring that the potential of technology is fully realized and not squandered, thus circumventing the risk of a burgeoning digital divide.

## 1. Introduction

Anemia and preeclampsia substantially threaten maternal health worldwide [1,2]. Anemia affects nearly 40% of pregnant women globally and carries severe risks, including preterm birth, low birth weight, and increased maternal mortality [3]. Similarly, preeclampsia—a hypertensive disorder of pregnancy [4]—is accountable for an alarming 14% of worldwide maternal deaths [5]. Malnutrition, in conjunction with poor health behavior, is one of the most significant factors responsible for anemia and preeclampsia among pregnant women [6,7,8,9,10,11,12] where they often suffer from imbalanced macro- and micronutrients, [13,14,15] not always due to lack of food but also due to wrong food choices, incorrect knowledge, and lack of nutrition literacy [16,17,18,19,20,21,22,23,24,25,26,27,28]. 

Notably, the burden of these conditions is disproportionately heavier in Low- and Middle-Income Countries (LMICs) such as India, where resources for prevention, detection, and management are often limited. India accounts for 17% of all maternal deaths worldwide [29], of which anemia contributes to more than 15% [7,30,31,32,33,34,35]. The alarming escalation in the prevalence of anemia among women in India is evident in the National Family Health Survey (NFHS-5, 2019–21) [36]. The data reveals a grave picture, with 57% of women aged 15–49 currently ((NFHS-5, 2019–21) suffering from anemia, marking a significant increase from the 53% recorded in the previous NFHS-4 survey (2015–16) [37]. Similarly, a study conducted in southern India reported a 32.60% prevalence of preeclampsia [38]. 

Several initiatives have been launched in India to address the severe impact of anemia and preeclampsia—these range from policy-making efforts to community-based approaches implemented by the government. Frontline Health Workers (FLHWs) have been appointed as part of these initiatives, and health centers have been established at the village level [39]. Despite concerted efforts to enhance community awareness through campaigns, supplemental counseling, and free nutrition supplementation, such as Iron Folic Acid tablets (IFA), these interventions have not achieved the desired results [40,41,42,43,44].

Could the challenges faced by traditional healthcare interventions in LMICs be overcome through a technological leap? Conventional community-based interventions often demand significant and ongoing fiscal and human resources, which may need to be more feasible and sustainable in LMICs [43,45]. Our previous work in rural India explored the potential of mobile health (mHealth) interventions and found promising results. We observed that these interventions significantly enhanced maternal health literacy, including HIV awareness, and stimulated positive behavioral changes such as adherence to recommended dosages of iron tablets [46,47]. Furthermore, we found that mHealth outperforms traditional community-based approaches [48]. Supporting our work, a three-month randomized controlled trial found smartphone-delivered intervention more effective than clinic-based group intervention [49]. Similarly, other studies have reported promising impacts of mHealth interventions on antenatal care quality, anemia management, nutrition consumption, and health behavior among pregnant mothers [50,51,52]. Evidence also shows the value of mobile health applications in managing preeclampsia among pregnant women within low-resource environments [53,54,55,56,57]. Further investigations in rural India have highlighted the significant role of such applications. The technology improved the capacity of community health workers (CHWs) to identify, manage, and counsel pregnant women at risk of preeclampsia and strengthen their interaction with communities [56]. 

In the face of compelling evidence supporting the effectiveness and sustainability of mobile health interventions, it is timely that governments and funding agencies in LMICs such as India embrace these promising technological solutions. Indeed, initial concerns around the digital divide and digital literacy may have been valid, but the fast-changing digital landscape is increasingly eclipsing these fears. Currently, mobile phone access in India stands at over 1.18 billion individuals [58,59]. Coupled with the expanding internet coverage, these developments form a powerful conduit for revolutionizing healthcare delivery through mobile health services [58,59,60,61]. Such technology not only makes health information and services more accessible but can also reach those living in the most remote areas, where traditional healthcare infrastructure often falls short.

Furthermore, many mobile applications targeting conditions such as anemia and preeclampsia have proven practical and user-friendly. Take the My Iron Friend app as an example: it has shown that monitoring iron levels can be an intuitive and accessible process, even for those without extensive technical knowledge [52]. Likewise, other apps have received accolades from users for their utility, user interfaces, and overall user experiences [50,51,56,62,63,64,65,66]. So, as we stand at the intersection of technological advancement and healthcare, the lack of mobile health interventions in India (or LMICs in general) appears increasingly unjustified. With the burgeoning access to mobile phones and the internet and the proven usability of health-related mobile applications, it is time we turn a new leaf in our approach. As the examples underscore, mobile health interventions harbor immense potential to tackle health concerns sustainably in a user-centric way, positioning them as invaluable allies in our quest to improve maternal health outcomes in LMICs.

In this exploratory study, we assess pregnant mothers’ perceptions of using mobile health interventions to manage conditions such as anemia and preeclampsia. The study explores whether mothers consider mobile health interventions beneficial for their health and well-being. Additionally, we also capture their health awareness and knowledge. 

## 2. Materials and Method

### 2.1. Ethics Statement 

This study received ethical approval from the West Virginia University Institutional Review Board under protocol number 2307814889. No personally identifiable information was collected during the study. Informed consent was obtained from all respondents before they initiated the survey. The consent form was attached to the survey. It included a comprehensive cover letter outlining the study’s purpose, the procedure involved, the approximate time to complete the survey, and assurances of anonymity and confidentiality. The cover letter also emphasized that participation was voluntary and that respondents could withdraw without consequences. The cover letter included contact information for the researchers in case respondents had any questions or concerns about the study. 

### 2.2. Study Site

The study was conducted in Jharkhand, India. According to the 2021 National Family Health Survey, about 67.5% of all expecting mothers in Jharkhand were anemic [36]. The state also suffers from preeclampsia and malnutrition [67,68]. Being a low-income state, Jharkhand also suffers from high maternal mortality and scarcity of healthcare services [69,70,71].

### 2.3. Health Technology Acceptance Model

To achieve the objective of this study, we combine the Health Belief Model (HBM) [72] and the Technology Acceptance Model (TAM) [73]. The HBM and the TAM are two theoretical frameworks that are used to understand and explain individuals’ acceptance and adoption of health-related technologies. The HBM was originally formulated to explain preventive health behavior [74]. It is based on the belief that individuals’ decisions to engage in health self-protection behaviors are influenced by perceived threat, outcome expectation, cues to action, and self-efficacy. The HBM proposes that individuals are more likely to adopt health-related technologies if they perceive a threat to their health, believe that the technology will be beneficial in reducing the threat, and have the confidence to use the technology effectively. On the other hand, the Technology Acceptance Model (TAM) focuses on individuals’ acceptance and use of information technology [75]. It posits that individuals’ acceptance of technology is influenced by two main factors: perceived usefulness and perceived ease of use. Perceived usefulness refers to the extent to which individuals believe that technology will enhance their performance or productivity, while perceived ease of use refers to the extent to which individuals believe that technology is easy to use. The TAM suggests that individuals are more likely to accept and use technology if they perceive it to be useful and easy to use. 

**Figure 1:**
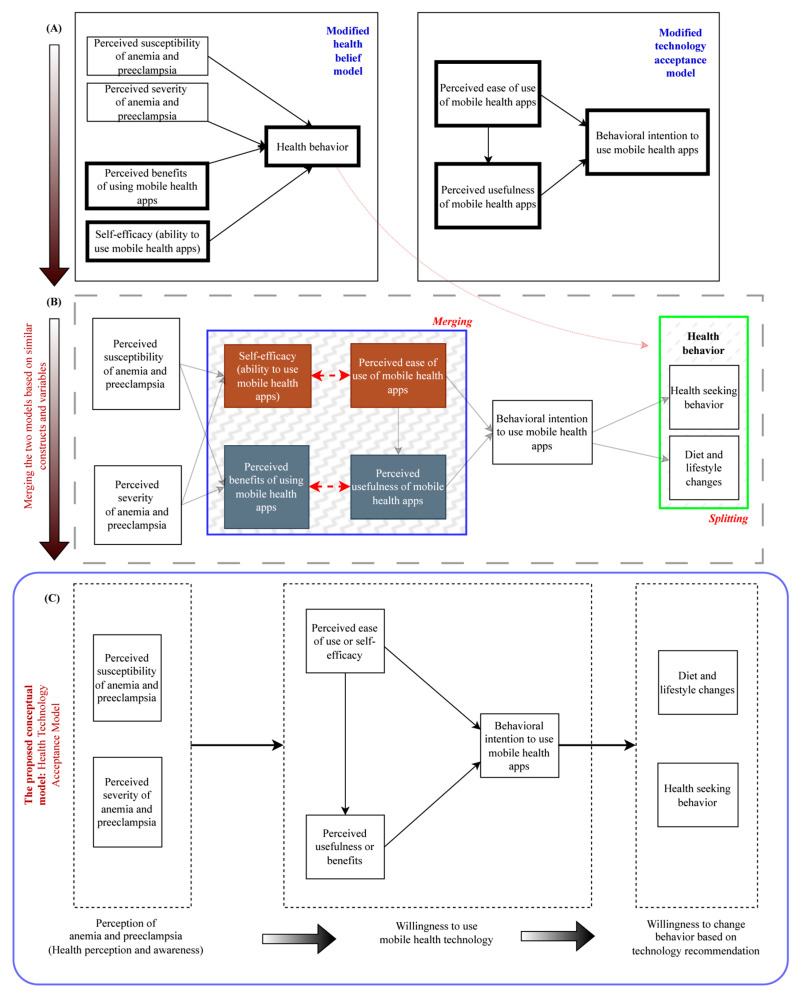
This figure shows the conceptual framework health technology acceptance model (HTAM). Panel (**A**) shows Health Belief (top left) and Technology Acceptance (top right) models. Panel (**B**) shows the merger of perceived ease of use with self-efficacy, perceived usefulness with perceived benefits, and behavior spitted into health-seeking behavior and diet and lifestyle changes. Panel (**C**) shows the proposed framework.

**Figure 2:**
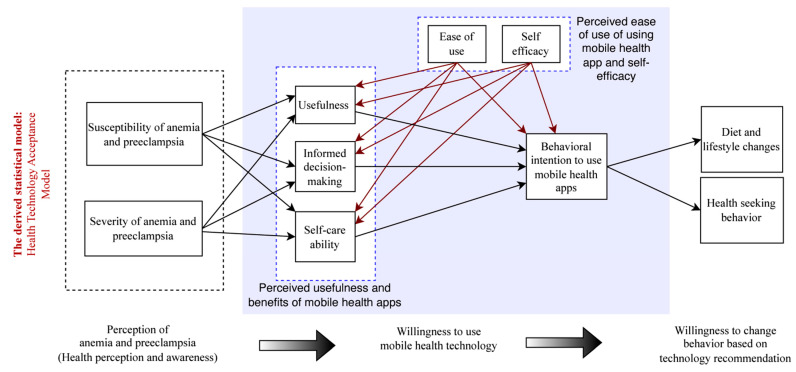
Exploratory statistical model.

Despite their differences in focus, the HBM and TAM share some similarities. Both models consider individuals’ perceptions and beliefs as important factors in determining their acceptance and adoption of technology. The HBM emphasizes the role of perceived threat and outcome expectation, while the TAM focuses on perceived usefulness and ease of use. Blending common elements from these two models, we created the Health Technology Acceptance Model (HTAM). Figure 1 illustrates the development of HTAM. Through the lens of our newly created HTAM, we explore multiple relationships, as illustrated in Figure 2.

### 2.4. Data Collection

**Figure 3:**
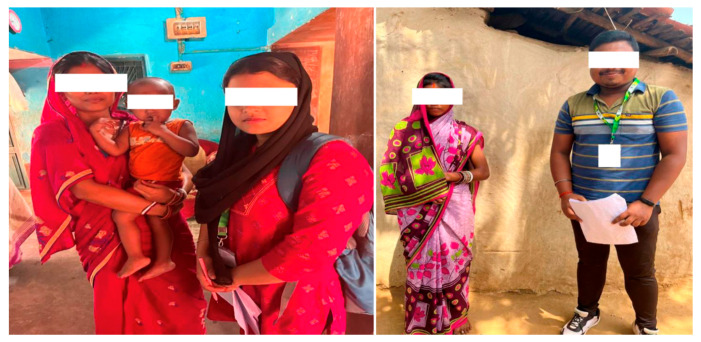
Field surveyor with respondents.

The data collection process was approached systematically. It was conducted in July 2023 by the Network for Enterprise Enhancement and Development Support (NEEDS), a non-governmental organization (NGO) based in India. Initially, we collaborated with our partnered NGO and the local village panchayat to identify households and make a list of all pregnant mothers in our target village. Next, we constituted five teams, each made up of a trained surveyor and an NGO field worker (see Figure 3). Their task was to survey three underserved villages in Jharkhand, India. Each team, responsible for visiting 6 to 8 villages, benefited from the familiarity and trust established by the NGO field worker within these communities, streamlining the survey process.

Upon reaching a household, the NGO field worker introduced the surveyor to the eligible mother. The surveyor then conveyed the study’s overall objective and the purpose of the survey in the local language. A cover letter detailing the study and data privacy measures was read to the mother from a tablet to obtain consent. The mother was then asked to click the ‘agree’ button on the tablet to signify her consent. With consent secured, the surveyor commenced the survey, reading each question aloud from the tablet in the local language and registering the mother’s response directly into the device. Mothers who were comfortable reading had the option to complete the survey independently. To create a comfortable and private environment, only the husband or a single older family member was permitted to be present during the survey, along with the research team. This measure was taken to ensure a conducive and respectful atmosphere for the respondents, reinforcing the ethical standards of our study. 

### 2.5. Survey Instrument

#### 2.5.1. Survey Development and Design

Our survey development process began with the design in English, using Qualtrics, an advanced online tool for creating and managing digital surveys. Since our target population was predominantly Hindi speaking, we prioritized translating the survey into Hindi to facilitate ease of understanding and response. The translation process was conducted meticulously, ensuring relevance and accuracy. The advantage of having native Hindi speakers among our team members (three authors) was instrumental in this translation task. Being fluent in both English and Hindi, they had the linguistic proficiency and cultural understanding to adapt the English survey into Hindi accurately while ensuring the integrity of the original questions remained intact. Following translation, the Hindi version of the survey was tested for clarity and ease of understanding. It underwent several iterations to fine-tune the language and ensure the translated questions accurately mirrored the intent and essence of the original English questions.

#### 2.5.2. Measuring Maternal Health Awareness and Knowledge

A comprehensive set of questions was incorporated into our survey to assess the respondents’ maternal health awareness and knowledge. These questions were designed to probe various aspects of maternal health, including nutrition, anemia, and preeclampsia.

Nutrition Awareness: respondents were asked about their overall knowledge of what to eat during pregnancy. To verify their knowledge, we included questions about specific nutrients essential fetal development and recommended foods for a healthy pregnancy diet.

Anemia Awareness: respondents were queried about their understanding of anemia, a common condition during pregnancy. We asked them about the primary cause of anemia and its effects on the health of the pregnant individual and their child. Additionally, we provided a list of common symptoms to ascertain their understanding of anemia symptoms and asked respondents to identify them.

Preeclampsia Awareness: respondents were asked about the primary cause of preeclampsia, its impact on the health of the pregnant individual and their child, and its common symptoms.

Each topic area was assessed through multiple-choice questions, with only one or multiple correct answers. The ‘Unsure’ option was provided for all questions to allow respondents to express uncertainty or lack of knowledge. 

#### 2.5.3. A Comprehensive Survey Based on Health Technology Acceptance Model

Table 1 lists the survey questions. Questions 1 to 6 were adapted from the Technology Acceptance Model (TAM) [76]. Questions 7 and 8 were adapted from Health Belief Model (HBM) [77]. Questions 9 and 10, about diet and lifestyle changes and health-seeking behavior, were used to assess the respondents’ intentions to change behavior based on mobile apps’ recommendations [78]. Responses were collected on a Likert scale. 

### 2.6. Statistical Analysis

All the analyses were performed using the SEMinR package [79] in R [80]. We analyzed our model using the non-parametric Partial Least Squares-Structural Equation Modeling (PLS-SEM) [74] approach. The model was controlled for age and education. The model fit was then evaluated by adjusted R-squared values [81]. We measure the significance of the model through the bootstrapping method with 10,000 subsamples [82], which involves resampling with replacement from the original sample to generate a new sample and subsequently estimating the model on the new sample. This process was repeated multiple times to produce a distribution of estimates, enabling more precise population inferences.

## 3. Results

### 3.1. Participant Characteristics

A total of 131 pregnant women completed the survey. Table 2 shows the participant characteristics. 

### 3.2. Smartphone Access

All the households had at least one smartphone. The survey results revealed that among the respondents, 44% (57 respondents) reported owning one smartphone per household. A considerable portion of households, 24% (32 respondents), owned two smartphones. About 3% (four respondents) owned three smartphones, and less than 1% (one participant) reported owning four. Regarding smartphone usage among household members, the respondents themselves made up the highest proportion at 47% (61 respondents). Spouses or partners were the next most frequent users, making up 29% (38 respondents) of smartphone users. The use of smartphones by children was reported by 4% (five respondents) of respondents, and less than 1% (one participant) reported parents using smartphones. Additionally, other members within the household were reported as smartphone users by 20% (26 respondents) of respondents.

### 3.3. Smartphone Use

The survey data indicates distinct usage patterns for the respondents and their household members when using smartphones. The most frequent use was for calling, reported by 24% (31 respondents). This was followed by text messaging, reported by 22% (29 respondents), and social media use, reported by 20% (26 respondents). Watching videos also represented a significant portion of smartphone use, reported by 13% (17 respondents). Interestingly, 21% (28 respondents) reported using their smartphones for other activities not specified in the survey. However, no respondents reported using their smartphones for health/fitness tracking.

### 3.4. Willingness to Use mHealth Apps

The prospect of a nutrition and pregnancy app was met with considerable interest among the surveyed respondents. A significant 61% (80 respondents) responded positively to the idea, indicating that they or someone in their household would be interested in such an application. Meanwhile, 15% (20 respondents) responded negatively, stating they would not be interested in the app. An additional 24% (31 respondents) were uncertain, indicating that they might be interested in the app, which suggests an opportunity for further exploration to understand their concerns or requirements.

### 3.5. Perceived Knowledge of Nutritional Requirements during Pregnancy

Regarding knowledge of appropriate dietary practices during pregnancy, 69% (90 respondents) reported having some knowledge. However, only 11% (15 respondents) claimed to have comprehensive knowledge. A significant 11% (14 respondents) admitted to having little to no knowledge, while 9% (12 respondents) were unsure about their level of understanding in this area.

### 3.6. Actual Knowledge of Nutritional Requirements during Pregnancy

Respondents were asked questions to assess their knowledge of dietary requirement during pregnancy. About 21% (28 respondents) accurately selected iron. No respondents correctly selected folic acid, a crucial nutrient for neural tube formation in the initial stages of pregnancy. Additionally, 70% (92 respondents) were unsure of the correct response, indicating a considerable knowledge gap. Concerning the foods recommended for a healthy pregnancy diet, the most accurately recognized food group was whole grains (e.g., brown rice, whole wheat bread, quinoa), with 78% (102 respondents) correctly identifying it as a beneficial part of a pregnancy diet. However, other recommended food groups such as fresh fruits and vegetables, lean proteins, low-fat dairy products, healthy fats, foods high in folic acid, and foods high in iron were only correctly identified by about 2% of respondents and 14% (18 respondents) were unsure of which foods are recommended for a healthy pregnancy diet.

### 3.7. Awareness of Anemia and Preeclampsia during Pregnancy

When asked about the primary cause of anemia and how it can impact the health of pregnant individuals and their babies, only 11% (14 respondents) correctly identified that a lack of iron is the primary cause and can lead to fatigue, complications during delivery, and low birth weight. Nearly 80% (105 respondents) reported being unsure of the correct answer. Furthermore, the survey explored respondents’ knowledge of common symptoms of anemia during pregnancy. The symptom most accurately recognized was shortness of breath, with 22% (29 respondents) correctly identifying it. Other common symptoms, including fatigue or weakness, pale skin, lips, and nails, rapid or irregular heartbeat, swollen or painful joints, frequent headaches, and dizziness or lightheadedness, were correctly identified by less than 7% of the respondents. About 60% (78 respondents) reported being unsure of the common symptoms of anemia.

The survey also examined respondents’ knowledge of preeclampsia, a serious pregnancy complication of high blood pressure. About 2% (three respondents) correctly identified that a placental disorder causes preeclampsia and can lead to premature birth, low birth weight, and complications for the mother, such as kidney damage and liver problems. Approximately 84% (110 respondents) reported being unsure about the primary cause of preeclampsia and its impact on the health of the pregnant individual and their child. Regarding the common symptoms of preeclampsia during pregnancy, the most accurately recognized symptom was swelling in the face, hands, and feet, correctly identified by 15% (19 respondents). However, other common symptoms, including high blood pressure, rapid weight gain over a short period, severe headaches, vision changes, nausea or vomiting, pain in the upper right side of the abdomen, and difficulty breathing or shortness of breath, were recognized by less than 8% of the respondents each. About 66% (87 respondents) reported being unsure of the common symptoms of preeclampsia.

### 3.8. Health Technology Acceptance Model

Before applying the bootstrapping method to ascertain statistical significance, the analysis delineates the correlation among different factors under study. In the context of perceived usefulness, the model specifies relationships with perceived severity (β = 0.416), perceived ease of use (β = 0.201), perceived susceptibility (β = 0.125), self-efficacy (β = 0.140), and education (β = 0.134), collectively explaining 54.1% of its variance (R^2^ = 0.541). When considering self-care ability, notable associations were detected with perceived ease of use (β = 0.393) and self-efficacy (β = 0.109), together accounting for 19.2% of the variance (R^2^ = 0.192). Informed decision-making displayed a correlation with self-efficacy (β = 0.323), perceived severity (β = 0.151), and perceived ease of use (β = 0.142), which cumulatively explained 31.8% of its variance (R^2^ = 0.318). Behavioral intention to use showed noteworthy associations with perceived usefulness (β = 0.332), self-care ability (β = 0.194), and perceived ease of use (β = 0.162), with a smaller correlation with self-efficacy (β = 0.068). The model accounts for 37.9% of the variance in behavioral intention to use (R^2^= 0.379). Lastly, behavioral intention to use exhibited a strong relationship between diet and lifestyle changes (β = 0.400) and health-seeking behavior (β = 0.533). These correlations account for 17.6% (R^2^ = 0.176) and 32.3% (R^2^ = 0.323) of the variance in each variable, respectively. Following the bootstrapping procedure, our analysis highlighted significant direct and total paths between study variables, as shown in Table 3. All other specific indirect paths are reported in the Supplementary File (see Table S1). 

**Figure 4:**
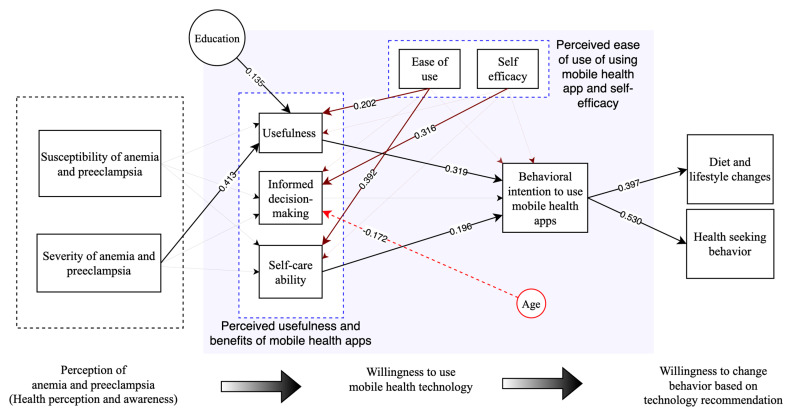
Illustration of the significant paths in the structural model. The values on the paths show the bootstrapped coefficients. The solid lines in the figure represent positive relationships, whereas the dotted lines represent inverse relationships.

The model indicates several implications. Firstly, a positive correlation exists between the perceived severity of anemia and preeclampsia and the usefulness of the mHealth applications. This suggests that pregnant women who view these health risks as severe also find mobile applications extremely beneficial in managing their health. Secondly, the perceived severity of health risks demonstrates a significant positive link to using mobile health apps, a willingness to adopt dietary and lifestyle modifications recommended by the apps, and proactive health-seeking behavior. This suggests that pregnant individuals who perceive these conditions as severe are more likely to use the application, adapt their lifestyle according to its advice, and seek medical attention. Consequently, these apps could be pivotal in promoting proactive health management behaviors among pregnant women, potentially leading to favorable health behavior modifications. Figure 4 illustrates the significant direct paths in the model.

## 4. Discussion

Our research extends the traditional Technology Acceptance Model (TAM) by integrating the Health Belief Model (HBM), culminating in a comprehensive Health Technology Acceptance Model (HTAM). This integrated model incorporates health-oriented factors, particularly perceived susceptibility, and severity, which encapsulate the user’s awareness of potential health risks and their magnitude. Our study findings significantly corroborate the effectiveness of HTAM, demonstrating substantial correlations between perceived susceptibility and severity of health conditions such as anemia and preeclampsia and the perceived usefulness of mobile health apps. These correlations suggest that an amplified awareness of health risks and their severity incline users to view health technology as beneficial, thereby boosting their acceptance rate. A key extension of the TAM in our study includes the behavioral intention to use mobile health apps, leading to the intent to modify health behaviors. This progression from technology acceptance to adopting healthier habits—such as dietary and lifestyle modifications and proactive health-seeking behavior—is a significant contribution. The positive pathways from perceived usefulness to behavioral intention and tangible health-related changes confirm this incorporation.

Our findings offer insights into how an application’s design and functionality influence user behaviors and health outcomes. For example, our study reveals that the perceived severity and the app’s perceived ease of use influence its usefulness. This suggests that users aware of potential health risks during pregnancy are more likely to find the app a valuable and user-friendly resource. Therefore, apps should incorporate educational content underlining the severe effects of conditions such as anemia on maternal and child health. The expectation-confirmation Model, a theoretical framework used to understand users’ continuance intention in information systems, suggests that users are more likely to continue using a system if they perceive it as useful [83]. 

Supporting our study, a study conducted a study on the factors influencing the intention to use self-diagnosis apps in Vietnam found that performance expectancy, effort expectancy, and hedonic motivation had a strong impact on users’ intentions to use apps [84].

The results of our study are revealing, pointing to a significant user readiness for the uptake of mobile health apps in the management of maternal health issues, such as anemia and preeclampsia, as well as in the sphere of nutrition. However, despite this apparent readiness, our research also shows that the application of mHealth technologies in these areas is far from being fully realized. An interesting observation is the widespread use of smartphones in households, with every household owning at least one and nearly half the respondents having an individual device. The usage, however, was limited to communication and social media, and surprisingly, none of the user employed smartphones for health or fitness tracking. This situation presents a missed opportunity and a ground for health-related interventions through mobile apps. 

Looking into the broader context, it is clear that our findings resonate with the existing body of research. For instance, a study focusing mainly on low-resource settings reveals that most mHealth initiatives are nascent, often limited to pilot projects centered around introducing and implementing new technologies [85]. The study underscores the necessity of a comprehensive approach in research that considers the various inputs, mechanisms, and outputs of mobile health interventions [85]. These findings suggest that the scope of interventions needs to be expanded beyond mere initial stages, focusing more on evaluating technology effectiveness and impact on maternal health outcomes. Moreover, another study discusses how mobile health innovations can play a pivotal role in enhancing maternal and child health in low-resource settings [86], emphasizing the need for context-specific interventions and sustainable, scalable solutions. 

Regarding willingness to use a mHealth app that centers on nutrition and pregnancy, most respondents expressed a keen interest. Similarly, a study in Africa on smartphone usage for weight management found a high willingness to use apps among women [87]. This underscores the potential receptivity of a mHealth application within this demographic, more so because most participants were personal smartphone users. However, some respondents exhibited uncertainty about their interest in such an app, highlighting a need for a deeper exploration of their hesitations or specific needs. 

The assessment of knowledge about nutrition during pregnancy revealed that while many participants claimed to have some understanding of dietary practices during pregnancy, only a handful professed comprehensive knowledge. More critically, a significant knowledge gap was exposed when questioned about the knowledge of nutritional needs during pregnancy. Similarly alarming findings were observed to understand pregnancy-related ailments such as anemia and preeclampsia. The data indicated a low level of correct identification of causes and impacts of these conditions, along with a low recognition rate of common symptoms. These findings stress the urgent need for educational initiatives to bolster knowledge about nutritional needs during pregnancy and pregnancy-related conditions. The widespread smartphone ownership and the generally positive attitude towards a mHealth app found in our study suggest that a mobile-based solution could potentially be a practical and effective strategy to address these concerns. 

Despite the noteworthy findings of our study, it has certain limitations. Firstly, it focuses solely on accepting mobile health technology amongst pregnant women, limiting the generalizability of the results to other demographic groups or different health contexts. Second, it largely relies on self-reported measures, which can introduce biases and may not necessarily correspond to actual behaviors. Third, we did not record any data to know if our participants had any miscarriage or have been pregnant earlier, as these may impact their perception and awareness regarding maternal health and nutrition. Fourth, we did not validate the self-reported information such as participant age, religion, education, proficiency in Hindi, and occupation. Furthermore, the cross-sectional nature of our study prevents us from making causal inferences or examining changes over time. Future research should address these limitations and continue to refine the HTAM for broader applicability.

## 5. Conclusions

Concurrent with the present study’s findings and the wider body of research within which it is framed, it is apparent that we stand at a pivotal moment where mobile health interventions can be systematically implemented to address critical maternal health issues in LMICs. With the proliferation of mobile phones and demonstrated receptiveness to mHealth applications, we are presented with a unique opportunity to catalyze significant advancements in this sphere. However, it is essential to act promptly. The potential drawback of delayed action could culminate in a digital divide, whereby despite available technology, a section of the population—in this context, mothers—remain unable to fully utilize its benefits due to the absence of suitable interventions and guidance. Thus, it is paramount to expediently establish comprehensive mHealth interventions to ensure inclusive and beneficial access to this digital evolution. Insights from this investigation, which probed into the prospective role of mHealth interventions among underprivileged Indian communities, offer compelling evidence supporting this claim. The study elucidated the escalating public health challenges of anemia and preeclampsia and highlighted a marked knowledge deficit. This finding underscores an urgent necessity for educational interventions—preferably facilitated by technology—to bridge this information gap. Notably, the study’s outcomes underscored a pronounced receptiveness of these women to mHealth applications specifically designed for managing anemia and preeclampsia. The Health Technology Acceptance Model (HTAM) deployed in this study illuminated the influence of the perceived severity of anemia and preeclampsia on the perceived ease of use and usefulness of mHealth interventions. It demonstrated how these perceptions consequently influenced their intention to use the app and their willingness to adopt health-promoting dietary and lifestyle changes, underscoring the potential efficacy of digital interventions within these communities. Furthermore, the study identified a positive correlation between higher levels of education and comfort with mobile phone usage and the perceived ease of use of mHealth applications. This finding indicates that as access to education and technology increases within these communities, the acceptance and practical utilization of such health-promoting apps will likely increase significantly. 

## Figures and Tables

**Table 1:** Survey questions and responses (*n* = 131).

Variable Names	Survey Questions		*n* (%)
Self-care ability (SA)	Q1. How likely is it that using a mobile health (mHealth) app for anemia and preeclampsia management would improve your ability to care for your health during pregnancy?	Extremely unlikely	6 (4.58)
		Unlikely	13 (9.92)
		Neutral	50 (38.17)
		Likely	43 (32.82)
		Extremely likely	19 (14.50)
Informed decision-making (IDM)	Q2. How useful do you think a mHealth app for anemia and preeclampsia management would be in helping you make informed decisions about your health during pregnancy?	Not useful at all	9 (6.87)
		Slightly useful	55 (41.98)
		Moderately useful	42 (32.06)
		Very useful	21 (16.03)
		Extremely useful	4 (3.05)
Perceived usefulness of mobile app (PU)	Q3. How effective do you believe using a mHealth app for anemia and preeclampsia management would be in reducing the risk of complications during pregnancy?	Not effective at all	15 (11.45)
		Slightly effective	41 (31.30)
		Moderately effective	43 (32.82)
		Very effective	30 (22.90)
		Extremely effective	2 (1.53)
Perceived ease of use of using mobile health apps (PEU)	Q4. How easy or difficult do you think it would be to use a mHealth app for anemia and preeclampsia management?	Extremely difficult	7 (5.34)
		Difficult	17 (12.98)
		Neutral	72 (54.96)
		Easy	30 (22.90)
		Extremely easy	5 (3.82)
Self-efficacy of using mobile health apps (SE)	Q5. How confident are you in your ability to use a mHealth app for anemia and preeclampsia management to improve your health during pregnancy?	Not confident at all	23 (17.56)
		Slightly confident	50 (38.17)
		Moderately confident	30 (22.90)
		Very confident	22 (16.79)
		Extremely confident	6 (4.58)
Behavioral intention to use mobile health apps (IU)	Q6. If a mHealth app for anemia and preeclampsia management were available, how likely would you be to use it during your pregnancy?	Extremely unlikely	7 (5.34)
		Unlikely	17 (12.98)
		Neutral	44 (33.59)
		Likely	56 (42.75)
		Extremely likely	7 (5.34)
Perceived susceptibility of anemia and preeclampsia (PSus)	Q7. How likely do you think it is for pregnant individuals to develop anemia and preeclampsia during pregnancy?	Extremely unlikely	7 (5.34)
		Unlikely	17 (12.98)
		Neutral	43 (32.82)
		Likely	57 (43.51)
		Extremely likely	7 (5.34)
Perceived severity of anemia and preeclampsia (PSer)	Q8. How serious do you think the consequences of anemia and preeclampsia are for pregnant individuals and their babies?	Not serious at all	17 (12.98)
		Slightly serious	55 (41.98)
		Moderately serious	39 (29.77)
		Very serious	18 (13.74)
		Extremely serious	2 (1.53)
Diet and lifestyle changes (DL)	Q9. If you were to use a mHealth app for anemia and preeclampsia management, how likely is it that you would make changes to your diet and lifestyle based on the app’s recommendations?	Extremely unlikely	10 (7.63)
		Unlikely	10 (7.63)
		Neutral	29 (22.14)
		Likely	54 (41.22)
		Extremely likely	28 (21.37)
Health-seeking behavior (HB)	Q10. After using a mHealth app for anemia and preeclampsia management, how likely would you be to seek medical advice or treatment for any symptoms or concerns related to anemia or preeclampsia?	Extremely unlikely	8 (6.11)
		Unlikely	10 (7.63)
		Neutral	37 (28.24)
		Likely	55 (41.98)
		Extremely likely	21 (16.03)

**Table 2:** Summary of the age distribution, employment, religion, level of education, and linguistic proficiency in Hindi of the 131 respondents.

Categories	Sub-Categories	Respondents *n*(%)
Age group (years)	25–34 years	72 (55)
	18–24 years	28 (21)
	35–44 years	28 (21)
	Under 18 years	1 (<1)
	45–54 years	2 (<1)
Employment	Homemakers	92 (70)
	Employed part-time	30 (23)
	Self-employed	8 (6)
	Unemployed	1 (<1)
Religion	Hindu	116 (89)
	Muslim	15 (11)
Level of education	Secondary (7–12 years)	57 (44)
	Primary (1–6 years)	27 (21)
	No formal education	28 (21)
	Bachelor’s degree	14 (11)
	Other	4 (<2)
Linguistic proficiency in Hindi	Speak, read, and write	48 (37)
	Speak only	30 (23)
	Speak and read	26 (30)
	No understanding	25 (19)
	Read only	2 (2)

**Table 3:** Direct and total path coefficients of study variables on health behavior determinants.

Paths	Direct Path			Total Path		
	β	SD	CI	β	SD	CI
PSus → PU	0.120	0.072	[−0.019, 0.261]	0.120	0.072	[−0.019, 0.261]
PSus → SA	0.025	0.096	[−0.163, 0.213]	0.025	0.096	[−0.163, 0.213]
PSus → IDM	0.080	0.095	[−0.107, 0.265]	0.080	0.095	[−0.107, 0.265]
PSus → IU				0.055	0.040	[−0.014, 0.143]
PSus → DL				0.023	0.018	[−0.005, 0.066]
PSus → HB				0.030	0.022	[−0.007, 0.082]
PSer → PU	0.413	0.075	[0.261, 0.552] *	0.413	0.075	[0.261, 0.552] *
PSer → SA	−0.173	0.089	[−0.353, 0.001]	−0.173	0.089	[−0.353, 0.000]
PSer → IDM	0.151	0.104	[−0.049, 0.359]	0.151	0.104	[−0.049, 0.359]
PSer → IU				0.120	0.061	[0.009, 0.247] *
PSer → DL				0.047	0.026	[0.003, 0.104] *
PSer → HB				0.063	0.032	[0.005, 0.129] *
PEU → PU	0.202	0.078	[0.050, 0.355] *	0.202	0.078	[0.050, 0.355] *
PEU → SA	0.392	0.097	[0.194, 0.574] *	0.392	0.097	[0.194, 0.574] *
PEU → IDM	0.139	0.103	[−0.068, 0.338]	0.139	0.103	[−0.068, 0.338]
PEU → IU	0.166	0.103	[−0.039, 0.363]	0.315	0.087	[0.139, 0.480] *
PEU → DL				0.127	0.051	[0.038, 0.237] *
PEU → HB				0.169	0.059	[0.061, 0.291] *
SE → PU	0.144	0.085	[−0.013, 0.322]	0.144	0.085	[−0.013, 0.322]
SE → SA	0.111	0.094	[−0.072, 0.295]	0.111	0.094	[−0.072, 0.295]
SE → IDM	0.316	0.113	[0.088, 0.527] *	0.316	0.113	[0.088, 0.527] *
SE → IU	0.075	0.096	[−0.107,0.268]	0.165	0.099	[−0.028, 0.358]
SE → DL				0.069	0.047	[−0.008, 0.172]
SE → HB				0.091	0.059	[−0.012, 0.216]
PU → IU	0.319	0.133	[0.053, 0.562] *	0.319	0.133	[0.053, 0.562] *
PU → DL				0.126	0.061	[0.019, 0.254] *
PU → HB				0.168	0.072	[0.028, 0.309] *
SA → IU	0.196	0.087	[0.026, 0.368] *	0.196	0.087	[0.026, 0.368] *
SA → DL				0.079	0.041	[0.009, 0.167] *
SA → HB				0.105	0.051	[0.013, 0.210] *
IDM → IU	0.089	0.112	[−0.130, 0.306]	0.089	0.112	[−0.130, 0.306]
IDM → DL				0.036	0.046	[−0.050, 0.128]
IDM → HB				0.048	0.060	[−0.066, 0.167]
IU → DL	0.397	0.096	[0.199, 0.577] *	0.397	0.096	[0.199, 0.577] *
IU → HB	0.530	0.083	[0.357, 0.683] *	0.530	0.083	[0.357, 0.683] *
Age → PU	0.004	0.062	[−0.118, 0.126]	0.004	0.062	[−0.118, 0.126]
Age → SA	−0.022	0.085	[−0.185, 0.144]	−0.022	0.085	[−0.185, 0.144]
Age → IDM	−0.172	0.074	[−0.315, −0.026] *	−0.172	0.074	[−0.315, −0.026] *
Age → IU	0.047	0.074	[−0.093, 0.197]	0.029	0.074	[−0.111, 0.179]
Age → DL	−0.095	0.088	[−0.271, 0.078]	−0.082	0.093	[−0.271, 0.094]
Age → HB	−0.003	0.079	[−0.163, 0.145]	0.012	0.085	[−0.161, 0.173]
Education → PU	0.135	0.060	[0.022, 0.256] *	0.135	0.060	[0.022, 0.256] *
Education → SA	0.089	0.097	[−0.097, 0.280]	0.089	0.097	[−0.097, 0.280]
Education → IDM	0.029	0.096	[−0.153, 0.222]	0.029	0.096	[−0.153, 0.222]
Education → IU	−0.035	0.104	[−0.229, 0.177]	0.031	0.117	[−0.190, 0.264]
Education → DL	0.066	0.095	[−0.116, 0.256]	0.078	0.099	[−0.111, 0.276]
Education → HB	0.123	0.082	[−0.029, 0.288]	0.138	0.093	[−0.038, 0.321]

β = bootstrapped standardized path coefficient; SD = bootstrapped standard deviation; CI = confidence interval; * significant at *p* < 0.05; SA = self-care ability (the mobile app will improve self-care ability); IDM = informed decision-making; PU = perceived usefulness of the mobile app; PEU = perceived ease of use of using mobile health apps; SE = self-efficacy of using mobile health apps; IU = behavioral intention to use mobile health apps; PSus = perceived susceptibility of anemia and preeclampsia; PSer = perceived severity of anemia and preeclampsia; DL = diet and lifestyle changes; HB = health seeking behavior.

## Data Availability

The data presented in this study are available on request from the corresponding author.

## Supplementary Materials

The following supporting information can be downloaded at: https://www.mdpi.com/article/10.3390/nu15173699/s1, Table S1: Indirect paths.
