# Supplementary material for: Exploring Perceptions and Needs of Mobile Health Interventions for Nutrition, Anemia, and Preeclampsia among Pregnant Women in Underprivileged Indian Communities: A Cross-Sectional Survey
Source: Nutrients. 2023 Aug 24;15(17):3699. doi: 10.3390/nu15173699 (PMC10490056; doi:10.3390/nu15173699)
Supplement: Supplementary file 1 [file nutrients-15-03699-s001.zip › nutrients-2559547-supplementary.pdf]

Table S1. Indirect paths

**Indirect effects of study variables**	**Original Est.**	**Bootstrap Mean**	**Bootstrap SD**	**T Stat.**	**CI [5%, 95%]**
Perceived susceptibility →Perceived usefulness→Behavioral intention to use →Health seeking behavior	0.022	0.021	0.017	1.268	[-0.002, 0.064]
Perceived susceptibility →Perceived usefulness→ Behavioral intention to use →Diet and lifestyle changes	0.017	0.016	0.014	1.204	[-0.002, 0.050]
Perceived susceptibility → Informed decision making, Behavioral intention to use →Diet and lifestyle changes	0.003	0.004	0.007	0.380	[-0.006, 0.022]
Perceived susceptibility → Informed decision making, Behavioral intention to use →Health seeking behavior	0.004	0.005	0.009	0.384	[-0.008, 0.030]
Perceived susceptibility → Selfcare ability, Behavioral intention to use →Diet and lifestyle changes	0.002	0.003	0.009	0.202	[-0.013, 0.024]
Perceived susceptibility → (Selfcare ability, Behavioral intention to use) →Health seeking behavior	0.002	0.003	0.012	0.209	[-0.018, 0.030]
Perceived severity →Perceived usefulness →Behavioral intention to use	0.138	0.136	0.067	2.049	[0.018, 0.274]
Perceived severity →Informed decision making →Behavioral intention to use	0.014	0.017	0.024	0.591	[-0.016, 0.077]
Perceived severity →Selfcare ability →Behavioral intention to use	-0.033	-0.032	0.022	-1.554	[-0.080, 0.003]
Perceived severity → (Perceived usefulness, Behavioral intention to use) →Diet and lifestyle changes	0.055	0.053	0.029	1.924	[0.006, 0.117]
Perceived severity → (Perceived usefulness, Behavioral intention to use) →Health seeking behavior	0.074	0.071	0.035	2.101	[0.009, 0.144]
Perceived severity → Informed decision making→ Behavioral intention to use →Diet and lifestyle changes	0.006	0.007	0.010	0.563	[-0.006, 0.033]
Perceived severity →Informed decision making→ Behavioral intention to use →Diet and lifestyle changes	0.008	0.009	0.013	0.571	[-0.008, 0.043]
Perceived severity → Selfcare ability→Behavioral intention to use→Health seeking behavior	-0.013	-0.013	0.009	-1.412	[-0.036, 0.001]
Perceived severity →Selfcare ability→ Behavioral intention to use →Diet and lifestyle changes	-0.018	-0.017	0.012	-1.464	[-0.045, 0.002]
Perceived ease of use →Perceived usefulness →Behavioral intention to use	0.067	0.063	0.035	1.896	[0.005, 0.141]
Perceived ease of use →Behavioral intention to use →Diet and lifestyle changes	0.065	0.067	0.046	1.416	[-0.013, 0.166]
Perceived ease of use →Behavioral intention to use →Health seeking behavior	0.087	0.089	0.058	1.492	[-0.019, 0.207]
Perceived ease of use → Perceived usefulness→ Behavioral intention to use →Diet and lifestyle changes	0.027	0.025	0.016	1.636	[0.002, 0.064]
Perceived ease of use → Perceived usefulness→ Behavioral intention to use →Health seeking behavior	0.036	0.033	0.020	1.810	[0.003, 0.078]
Perceived ease of use →Selfcare ability →Behavioral intention to use	0.076	0.077	0.040	1.906	[0.009, 0.165]
Perceived ease of use → Selfcare ability→ Behavioral intention to use →Diet and lifestyle changes	0.030	0.031	0.019	1.615	[0.003, 0.075]
Perceived ease of use → Selfcare ability→ Behavioral intention to use →Health seeking behavior	0.041	0.041	0.024	1.724	[0.004, 0.095]
Self-efficacy →Perceived usefulness →Behavioral intention to use	0.047	0.043	0.030	1.567	[-0.007, 0.110]
Self-efficacy →Behavioral intention to use →Diet and lifestyle changes	0.027	0.033	0.042	0.654	[-0.036, 0.126]

Self-efficacy →Behavioral intention to use →Health seeking behavior	0.036	0.043	0.054	0.677	[-0.050, 0.159]
Self-efficacy → Perceived usefulness→ Behavioral intention to use → Diet and lifestyle changes	0.019	0.017	0.013	1.379	[-0.002, 0.050]
Self-efficacy → Perceived usefulness→ Behavioral intention to use →Health seeking behavior	0.025	0.023	0.017	1.459	[-0.003, 0.064]
Self-efficacy →Informed decision making →Behavioral intention to use	0.030	0.026	0.038	0.793	[-0.049, 0.108]
Self-efficacy →Informed decision making→ Behavioral intention to use →Diet and lifestyle changes	0.012	0.010	0.015	0.810	[-0.018, 0.043]
Self-efficacy →Informed decision making→ Behavioral intention to use →Health seeking behavior	0.016	0.014	0.020	0.806	[-0.024, 0.058]
Self-efficacy →Selfcare ability →Behavioral intention to use	0.021	0.021	0.022	0.970	[-0.015, 0.071]
Self-efficacy →Selfcare ability→ Behavioral intention to use →Diet and lifestyle changes	0.008	0.008	0.009	0.904	[-0.006, 0.031]
Self-efficacy →Selfcare ability→ Behavioral intention to use →Health seeking behavior	0.011	0.011	0.012	0.929	[-0.008, 0.040]
Self-efficacy →Behavioral intention to use →Diet and lifestyle changes	0.027	0.033	0.042	0.654	[-0.036, 0.126]
Self-efficacy →Behavioral intention to use →Health seeking behavior	0.036	0.043	0.054	0.677	[-0.050, 0.159]
Perceived usefulness →Behavioral intention to use →Diet and lifestyle changes	0.133	0.126	0.061	2.185	[0.019, 0.254]
Perceived usefulness →Behavioral intention to use →Health seeking behavior	0.177	0.168	0.072	2.458	[0.028, 0.309]
Informed decision making →Behavioral intention to use →Diet and lifestyle changes	0.038	0.036	0.046	0.824	[-0.050, 0.128]
Informed decision making →Behavioral intention to use →Health seeking behavior	0.050	0.048	0.060	0.832	[-0.066, 0.167]
Selfcare ability →Behavioral intention to use →Diet and lifestyle changes	0.077	0.079	0.041	1.870	[0.009, 0.167]
Selfcare ability →Behavioral intention to use →Health seeking behavior	0.103	0.105	0.051	2.016	[0.013, 0.210]
